# Glucagon-Like Peptide-1 Receptor Agonists and Risk of Adverse Maternal Pregnancy Outcomes: A Systematic Review and Meta-analysis

**DOI:** 10.1097/AOG.0000000000006363

**Published:** 2026-07-02

**Authors:** Erin Hattler, Hannah Schluter, Catherine Greene, Margaret A. Adgent, Alexandra C. Sundermann, Elizabeth A. Jasper

**Affiliations:** Vanderbilt University, and the Department of Health Policy, the Division of Quantitative and Clinical Sciences, Department of Obstetrics and Gynecology, and the Department of Biomedical Informatics, Vanderbilt University Medical Center, Nashville, Tennessee.

## Abstract

**OBJECTIVE:**

To assess associations between pregestational and early-gestational exposure to glucagon-like peptide-1 receptor agonists (GLP-1 RAs) and maternal pregnancy complications.

**DATA SOURCES:**

A comprehensive search across the PubMed and EMBASE databases was conducted from inception to November 2025.

**METHODS OF STUDY SELECTION:**

Eligibility criteria for inclusion were 1) exposure to GLP-1 RAs before or during gestation; 2) cohort, case–control, or randomized controlled trial (RCT) study reporting quantitative data on maternal obstetric outcomes; and 3) study population greater than 10. Two reviewers independently abstracted study data and assessed quality and risk of bias using the Newcastle–Ottawa Quality Assessment Scale for observational studies and the Risk of Bias for Randomized Crossover Trials tool for RCTs. Odds ratios (ORs) were pooled using random effects with the Knapp–Hartung adjustment to reduce chance of false-positives and the restricted maximum likelihood estimator for heterogeneity testing.

**TABULATION, INTEGRATION, AND RESULTS:**

Eight studies totaling 186,598 pregnancies (47,159 exposed) were included. No statistically significant differences were seen for gestational diabetes (OR 0.99, 95% CI, 0.61–1.61), preterm birth (OR 1.01, 95% CI, 0.76–1.33), preeclampsia (OR 1.05, 95% CI, 0.60–1.84), or hypertensive disorder of pregnancy (OR 0.79, 95% CI, 0.34–1.83), although results from leave-one-out sensitivity testing suggest that GLP-1 RA exposure may have a protective effect against developing gestational diabetes (OR 0.81, 95% CI, 0.67–0.98). Newcastle–Ottawa Quality Assessment Scale results demonstrated variability in study quality and high heterogeneity attributable to differences in exposure and outcome definitions, cohort selection, and control for confounders.

**CONCLUSION:**

Use of a GLP-1 RA in the peri-fertilization period was not associated with change in odds of maternal pregnancy complications. Exploratory sensitivity results suggest that peri-fertilization exposure may lower odds of gestational diabetes. Further research is necessary to explore these hypotheses-generating results.

## Abstract 2

Existing literature suggests no association between peri-fertilization glucagon-like peptide-1 receptor agonist exposure and adverse maternal outcomes. Further research is necessary given scarce and heterogeneous prior studies.

In 2019, an estimated 29% of pregnancies were affected by prepregnancy overweight or obesity,^1,2^ and about 79 million reproductive-aged women were affected by diabetes in 2021.^3–5^ Obesity is a risk factor for poor health outcomes, a major driver of subfertility, and one of the greatest modifiable risk factors for adverse pregnancy outcomes.^6–8^ Elevated prepregnancy body mass index (BMI, calculated as weight in kilograms divided by height in meters squared) is associated with hypertensive disorders of pregnancy (HDP), gestational diabetes mellitus (GDM), preterm or stillbirth, inappropriate gestational weight gain, unscheduled cesarean delivery, venous thromboembolism, and postpartum wound complications.^6,8–12^ Elevated blood glucose levels can further increase the risk of pregnancy complications, including spontaneous abortion, preeclampsia, and congenital anomalies.^12–17^

Prepregnancy weight loss has been shown to improve glycemic control, lower the risk of adverse pregnancy outcomes, and improve fertility.^7,18–22^ Optimization of BMI is recommended for individuals with overweight or obesity planning pregnancy.^6,8,10,23^ Although lifestyle modifications and bariatric surgery have long been mainstays of obesity treatment, in recent years, glucagon-like peptide-1 receptor agonists (GLP-1 RAs) have gained popularity.^24–26^ As weight loss medications, GLP-1 RAs are notable for their efficacy and safety profile.^24,26,27^ They have been shown to improve cardiometabolic parameters and glycemic control in individuals with type 2 diabetes^28^ and to positively affect hormonal regulation and fertility in individuals with polycystic ovarian syndrome (PCOS).^19,23,29^

Between 2017 and 2021, there was an estimated 40-fold increase in GLP-1 RA use.^30^ Clinical guidelines recommend stopping GLP-1 RAs before pregnancy because of insufficient data on the effects on pregnancy and offspring^7,31,32^ However, as GLP-1 RA use has risen, so has the number of pregnancies with unintentional exposure.^14,33–35^ Although research has been reassuring regarding risk of birth defects after inadvertent first-trimester exposure,^14,33–36^ data on maternal obstetric outcomes have conflicting,^7,32,37^ suggesting potential for maternal benefit^38–40^ or harm.^40,41^ The true direction and strength of associations remain unknown.

The aim of this systematic review and meta-analysis is to assess the current state of the research on the association between peri-fertilization exposure to GLP-1 RAs and subsequent pregnancy outcomes. We aimed to answer the following questions:

1. What is known about maternal obstetric outcomes after *peri-fertilization exposure* (for the purpose of our analysis broadly defined as exposure before and into pregnancy) to GLP-1 RAs?
2. What are the gaps in literature?
3. What is the overall heterogeneity in study design, and how can research methods be improved moving forward?

## SOURCES

This study adhered to the MOOSE (Meta-analysis of Observational Studies in Epidemiology) reporting guidelines for systematic reviews and meta-analyses.^42^ A systematic literature search across the PubMed and EMBASE databases was conducted from their inception through November 25, 2025. Key words used in the search included “glucagon-like-peptide-1,” “GLP-1 RA,” “prepregnancy,” “preconception,” “maternal outcomes,” “hypertensive disorder of pregnancy,” “gestational diabetes,” and “preeclampsia.” Search strategy was customized by database (Appendix 1, available online at http://links.lww.com/AOG/E735). Reference lists of identified studies were manually checked for additional relevant articles.

## STUDY SELECTION

Inclusion eligibility criteria were the following: 1) study population that included women with and without exposure to GLP-RAs before or during pregnancy; 2) original cohort, case–control, or randomized controlled trial (RCT) study reporting quantitative data on obstetric outcomes; and 3) study population greater than 10. Our main outcomes were GDM, HDP, preeclampsia, and preterm birth (PTB). Secondary outcomes included birth by cesarean delivery, miscarriage, and gestational weight gain. We intentionally kept the prepregnancy exposure period broad to ensure that we captured all existing literature.

Studies were excluded if they lacked adequate quantitative information for effect measure estimation or if their study population overlapped with that of another included study. Only human studies written in English were included. Case reports or series, commentaries, cross-sectional studies, and duplicate studies were excluded. We did not include any conference abstracts, graduate theses, or articles that were not peer reviewed because the systematic literature review did not identify any gray literature meeting other inclusion criteria.

Titles and abstracts of sourced articles were assessed for relevance. Two independent reviewers (E.H. and H.S.) reviewed full texts of relevant articles for eligibility, abstracted study data, and assessed quality through a standardized abstraction protocol using REDCap.^43^ A third reviewer (C.G.) evaluated abstracted data for discrepancies between reviewers. Consensus was reached through group discussion.

Data abstracted from studies encompassed study characteristics (authors, publication year, study period, country, study design, inclusion and exclusion criteria, sample size), participant characteristics (age, prepregnancy BMI or weight, comorbidities, obstetric history, and medications), and statistical analysis methods. Details about the exposure and outcomes (definitions, exposure indication and timing, assessment methods, specific GLP-1 RA studied, and follow-up periods) were also collected.

Most studies reported effects as odds ratios (ORs) or risk ratios. If a study reported both adjusted and unadjusted measures, the adjusted measure was used for the meta-analysis. For studies reporting risk ratios or without effect measures, reported raw numbers were used to calculate unadjusted ORs. Effect estimates were pooled to obtain a summary effect estimate using random-effects modeling, with the restricted maximum likelihood method for heterogeneity variance (τ^2^) and Hartung–Knapp adjustments to calculate 95% CIs and 95% prediction intervals around the pooled effect. Prediction intervals describe the estimated range of true effect sizes for each outcome and aid in interpretation of heterogeneity. The Hartung–Knapp adjustment reduces chances of a false-positive result when the study number is low or there are large differences in sample size.^44–47^ Analyses were completed with R 4.5,^48^ Meta 8.2–1,^49^ and Metafor 4.8–0^50,51^ packages. Robustness was assessed by leave-one-out analysis for outcomes with at least four studies; further sensitivity testing through graphical display of study heterogeneity diagnostics and subgroup analysis was reserved for those with more than five.^52^ Statistical tests for publication bias were not performed because of inadequate power.^45,50^ Results were considered statistically significant if *P*<.05.

The Newcastle–Ottawa Quality Assessment Scale^53^ was used to evaluate risk of bias for observational studies, evaluating selection, comparability, and assessment of outcomes. Studies could receive a maximum score of 9; a score of 8 or higher was considered high quality. Studies with a score of zero for comparability were automatically rated as poor quality. The RoB 2 (Revised Cochrane Risk of Bias for Randomized Crossover Trials) tool^54^ was used to evaluate potential bias for RCTs. Studies with a high risk of bias were noted, and their influence on findings was considered during data interpretation.

## RESULTS

**Fig. 1.**
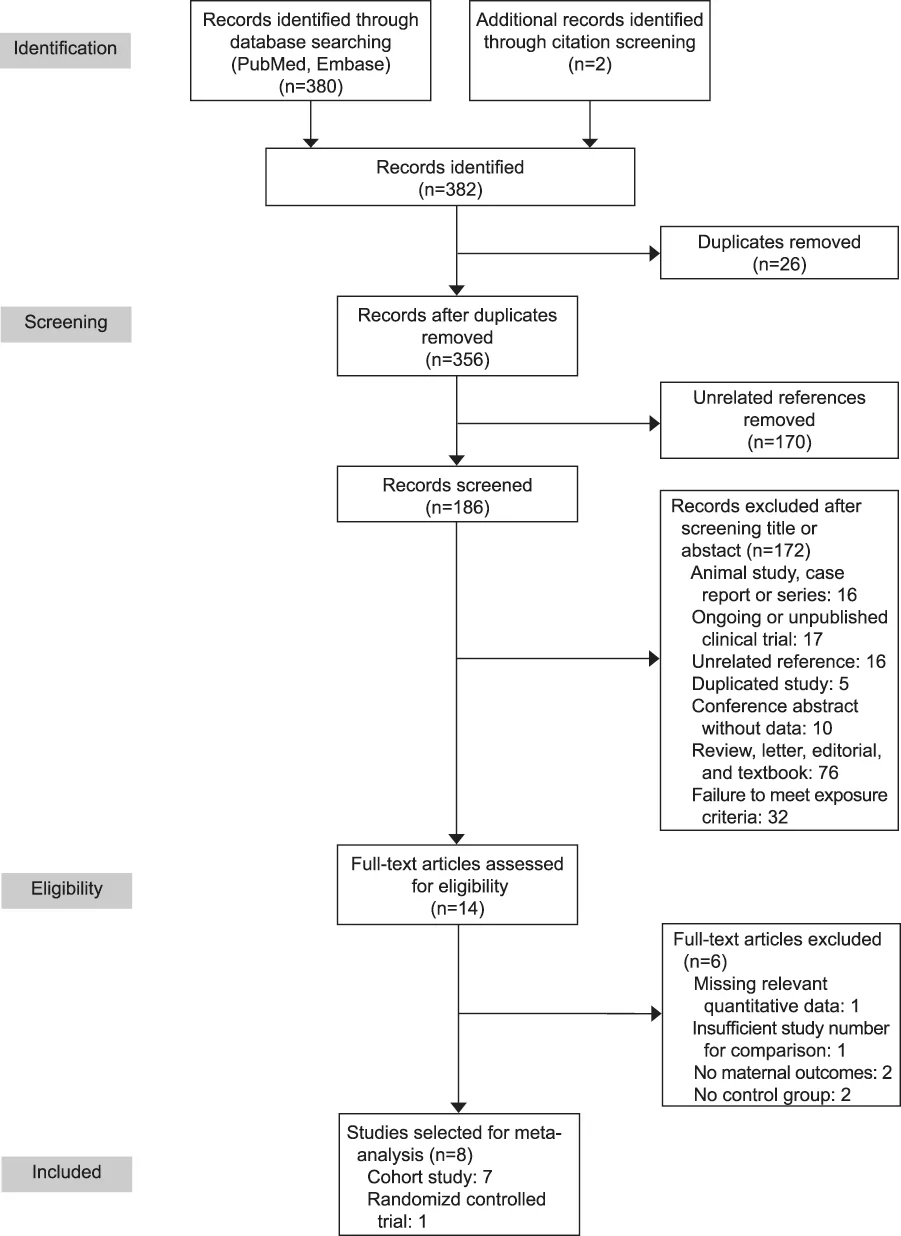
Study flow diagram.

We identified 380 records for screening. Two additional studies were identified through citations. After duplicates were removed, 356 records remained. A total of eight studies were eligible for meta-analysis after inclusion and exclusion criteria were applied (Fig. 1).

*Hattler. GLP-1 RAs and Pregnancy Outcomes Meta-analysis. Obstet Gynecol 2026*.

The meta-analysis included seven observational cohort studies^35,38–41,55,56^ and one long-term follow-up of an RCT.^57^ More than 186,500 pregnancies were meta-analyzed: 47,159 with prepregnancy or gestational exposure to GLP-1 RAs and 139,439 without (Table 1). Studies varied significantly in design. The considerable variability in outcome definitions and small number of studies precluded a pooled analysis of gestational weight gain, miscarriage, and cesarean delivery. Studies represented primarily North American and Western European populations, although the Li et al^57^ study was conducted in China and Hanif et al^39^ used a global database. Most studies noted baseline differences in exposed compared with unexposed cohorts.^35,38–41,55^ In general, GLP-1 RA users were older and had higher BMI, public insurance, and a greater burden of comorbidities. The Maya et al^41^ prematch exposure cohort was the exception; patients had lower mean BMI, were younger, and had fewer comorbidities.

**Table 1.**
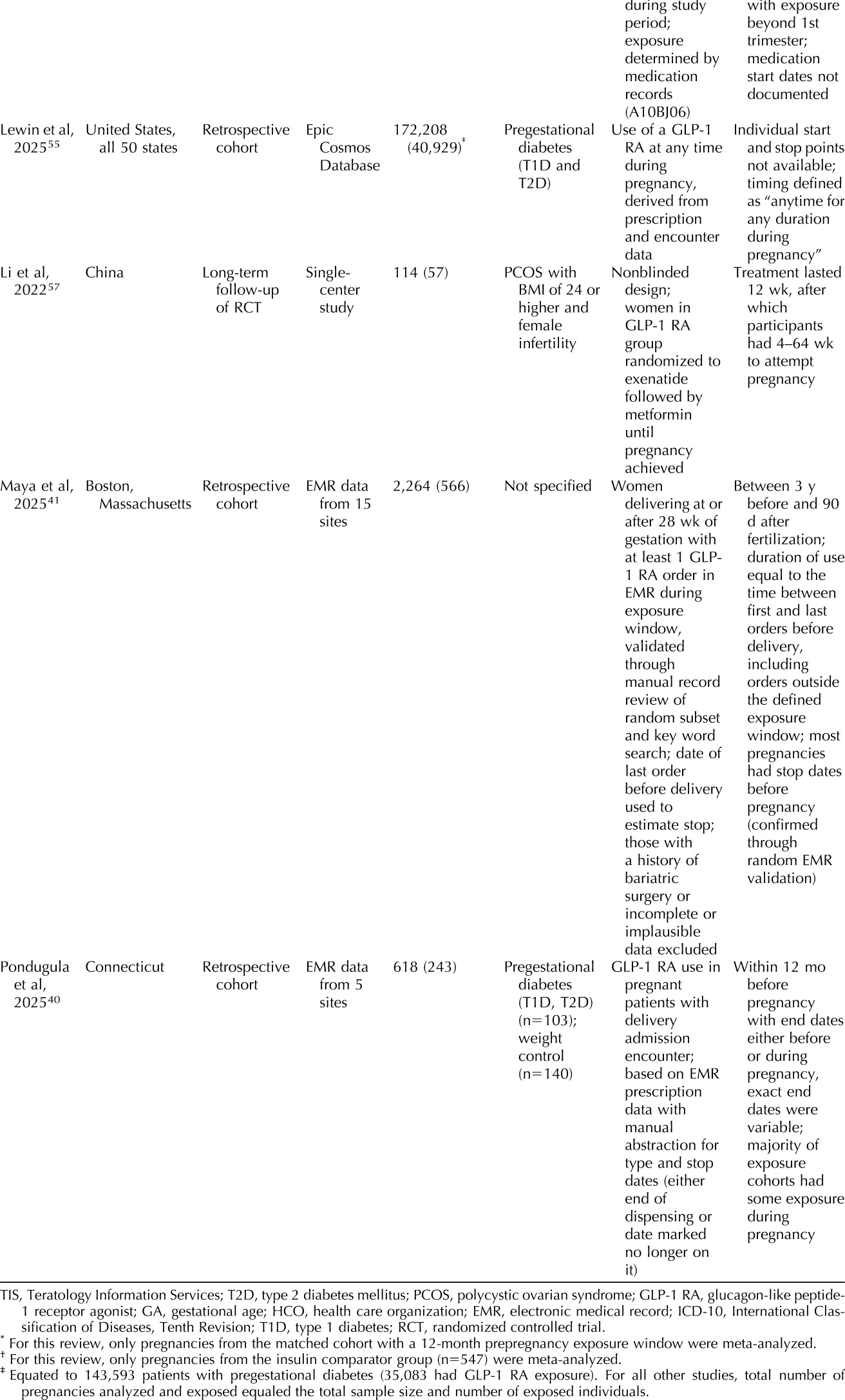
Study Characteristics and Exposure Details

There was variation in study population selection criteria. Some studies grouped exposed by indication (ie, weight loss management vs pregestational diabetes), and others pooled all exposed into a single group. Choice of exposure cohort influenced selection of unexposed. In studies using pooling, unexposed included anyone without GLP-1 RA use and typically matched or adjusted for elevated BMI and medical conditions. For those using grouping, unexposed comparators typically were selected according to the presence of a similar indicating condition (ie, prepregnant with obesity or overweight not on a GLP-1 RA, pregestational diabetes managed with a non–GLP-1 RA antihyperglycemic medication). Kolding et al^56^ used two unexposed comparison groups, one population-based and unmatched, one smaller and matched by pregestational diabetes status. To improve comparability, we used effect estimates from the latter in our analysis.

Exposure assessment windows and definitions were inconsistent across studies. Although all observational studies identified exposures using prescription data and Anatomical Therapeutic Chemical classification codes, only four^35,40,41,56^ used manual chart review, interviews, or pharmacy dispensation data to cross-validate exposures. Most studies included all available GLP-1 RA medications; however, two^56,57^ limited to one medication. Three studies^35,55,56^ used GLP-1 RA exposure definitions that required use in pregnancy and provided limited characterization of prepregnancy exposure. Five^38–41,57^ assessed prepregnancy exposures; four^38–41^ also continued into pregnancy. Most prepregnancy exposure windows were defined as within 12 months of fertilization. The Maya et al study*,*^41^ in which the exposure window was within 36 months, was the exception but reported that 66% of exposed pregnancies included exposures within 6 months of fertilization. The Li et al study,^57^ the only RCT, followed up women attempting pregnancy after a 12-week exposure period, and Imbroane et al^38^ assessed prepregnancy exposures within four cohorts (24, 12, 6, and 3 months). Our meta-analysis used the 12-month cohort to improve comparability with other studies. Only three studies^40,41,57^ could confirm exact exposure end points. Outcomes were defined as binary variables and determined primarily using the presence of International Classification of Disease, Tenth Revision (ICD-10) codes (Table 2), although coding definitions varied. The Maya et al^41^ study was the only cohort study to include laboratory values and objective measurements in their definitions. Kolding et al^56^ censored numbers less than 10, with the number of GLP-1 RA–exposed individuals experiencing GDM appearing as less than three. For analyses, we input two for the exposed GDM cases.

**Table 2.**
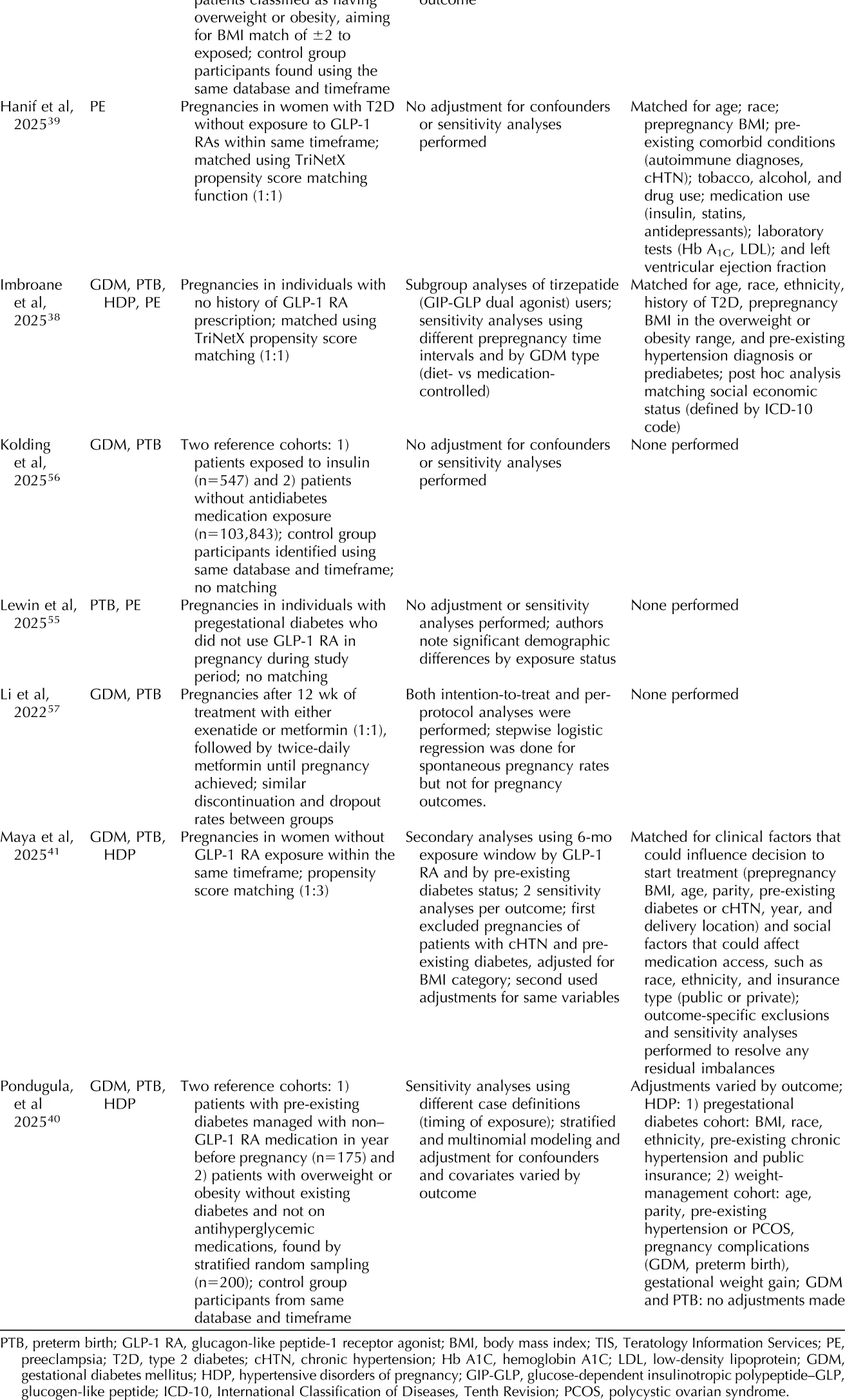
Included Outcomes, Control Selection, and Statistical Methods

**Fig. 2.**
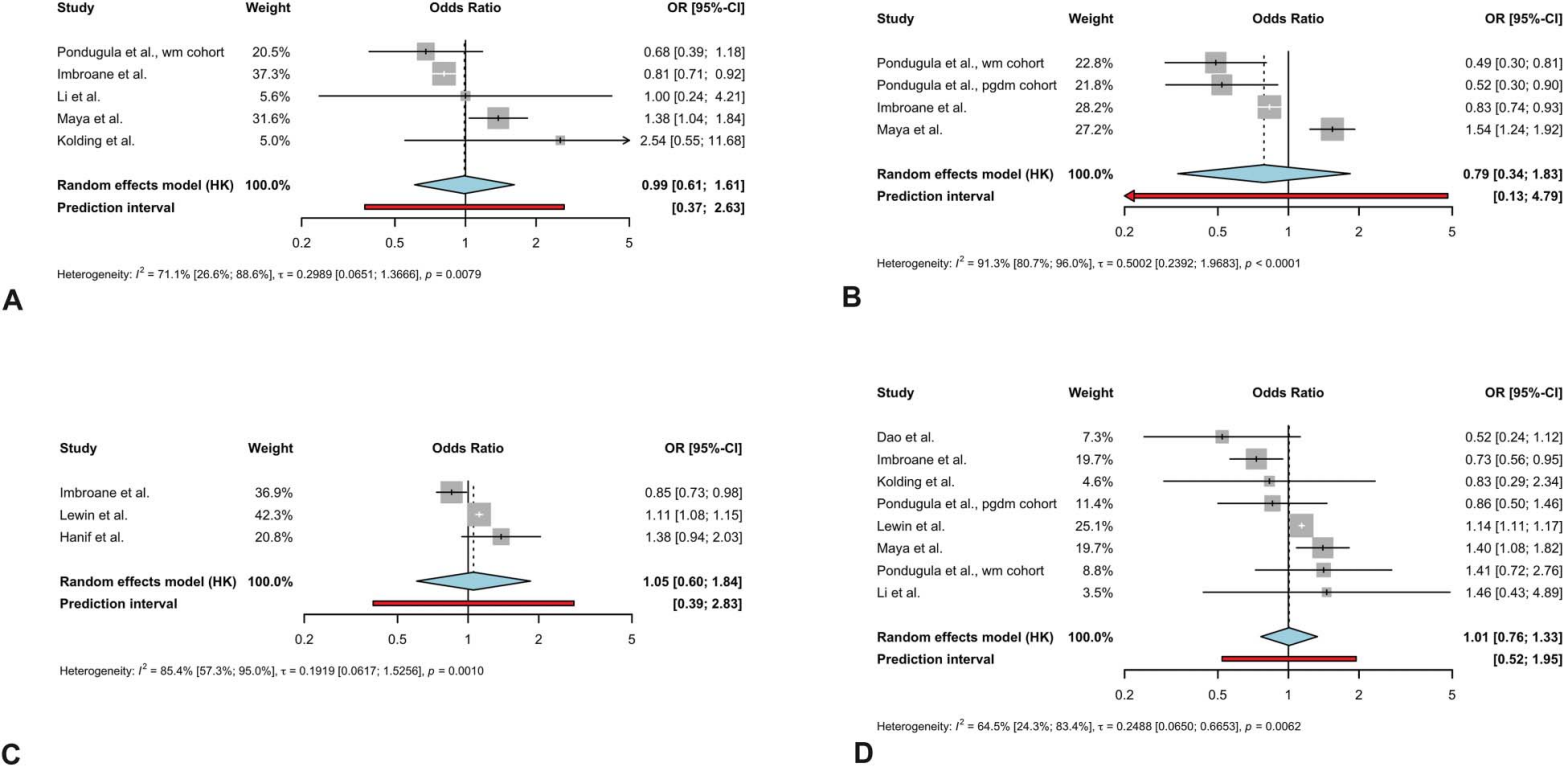
Forest plots of meta-analyses examining associations between glucagon-like peptide-1 receptor agonist (GLP-1 RA) exposure and maternal pregnancy outcomes. Gestational diabetes mellitus **(A)**, hypertensive disorders of pregnancy **(B)**, preeclampsia **(C)**, and preterm birth **(D)**. *Squares* represent individual study effect sizes with 95% CIs, with square size proportional to study weight. *Diamonds* represent pooled effect estimates with 95% CIs. *I*^*2*^ indicates heterogeneity; τ^2^ represents between-study variance. OR, odds ratio.

**Fig. 3.**
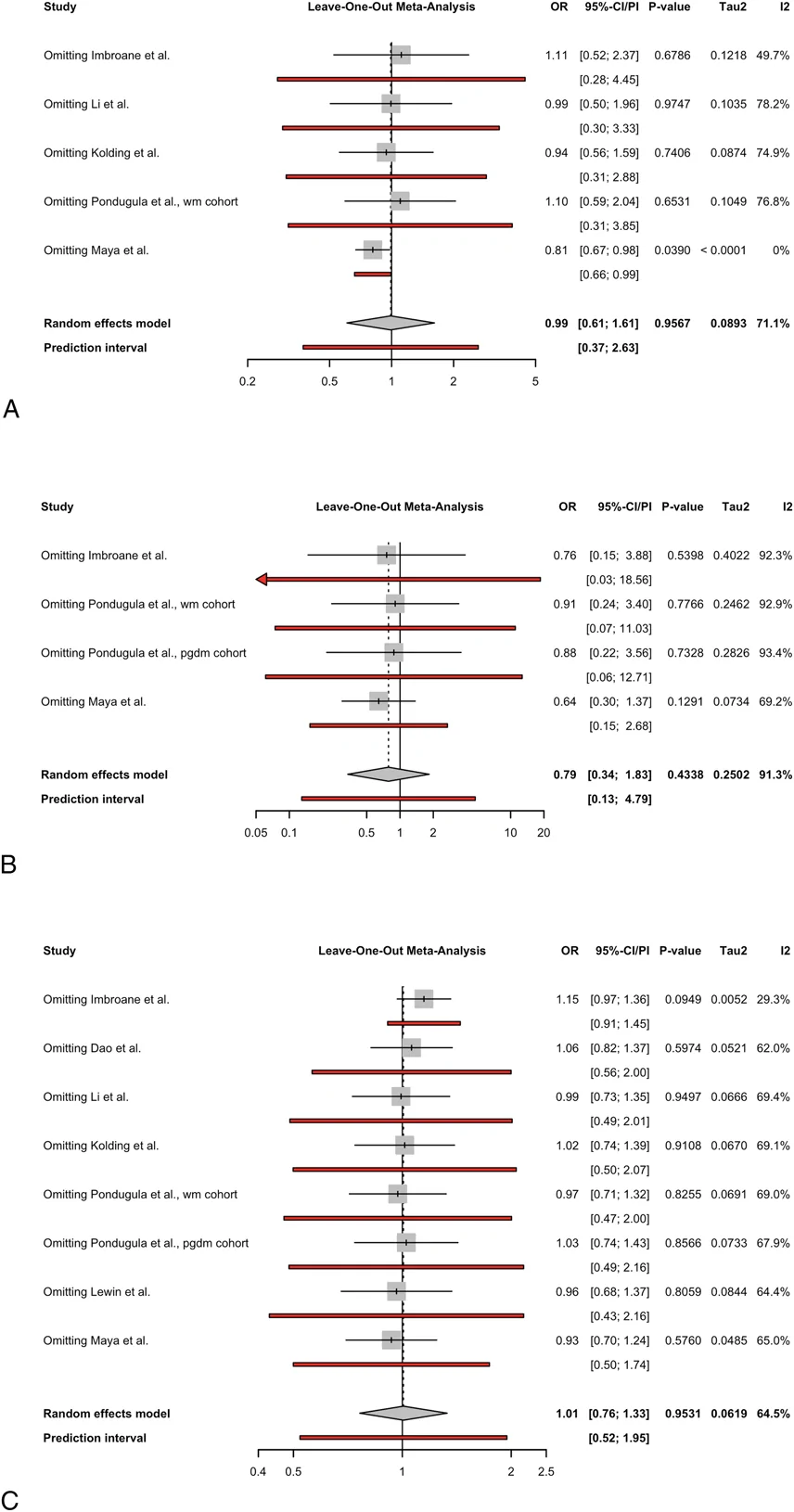
Leave-one-out sensitivity analysis for the association between exposure and gestational diabetes mellitus **(A)**, hypertensive disorders of pregnancy **(B)**, and preterm birth **(C)**. Each *row* represents the pooled effect estimate with the corresponding study omitted. Pooled estimates were derived using a random-effects model with restricted maximum likelihood estimation and Hartung–Knapp adjustment. *Diamonds* represent the overall pooled estimate; *red lines* represent 95% prediction intervals. OR, odds ratio.

Five studies^38,40,41,56,57^ representing 9,354 pregnancies (3,973 with GLP-1 RA exposure and 5,381 without) were meta-analyzed to examine the association between peri-fertilization GLP-1 RA use and GDM (Fig. 2A). Four^38,40,41,57^ assessed prepregnancy exposures. The pooled effect was not significant (OR 0.99, 95% CI, 0.61–1.61). Heterogeneity was significant, with a between-study heterogeneity variance of τ^2^=0.09 (95% CI, 0.00–1.87), SD of true effect sizes^45^ of τ=0.30, and an *I*^2^ value of 71.1% (*P*=.008). The OR prediction interval^58^ ranged from 0.37 to 2.63, indicating a wide range of expected true effects based on included studies. Leave-one-out sensitivity analysis indicated that the Maya et al^41^ study was a major contributor to heterogeneity (Fig. 3A). When omitted, results were statistically significant with decreased odds of developing GDM for women with GLP-1 RA exposure (OR 0.81, 95% CI, 0.67–0.98) and low or insignificant heterogeneity (*I*^2^=0%, *P*=.45).

*Hattler. GLP-1 RAs and Pregnancy Outcomes Meta-analysis. Obstet Gynecol 2026*.

*Hattler. GLP-1 RAs and Pregnancy Outcomes Meta-analysis. Obstet Gynecol 2026*.

Numerous factors contributed to study heterogeneity. Although pregestational diabetes should preclude a GDM diagnosis, all but one^56^ improved validity by matching or adjusting on this variable. Outcome assessment varied. Two studies^41,57^ used the Association of Diabetes and Pregnancy Study Groups diagnostic criteria^59^ as their primary measure, and the others^38,40,56^ used ICD codes exclusively.

With regard to HDP, three studies with four distinct cohorts^38,40,41^ representing 9,015 pregnancies (4,006 exposed and 5,009 unexposed) were eligible. All included prepregnancy exposures. Results showed decreased odds of developing an HDP after peri-fertilization exposure to GLP-1 RAs but were not significant (OR 0.79, 95% CI, 0.34–1.83) (Fig. 2B). There was significant, high between-study heterogeneity (τ^2^=0.25, 95% CI, 0.06–3.87, τ=0.50, *I*^2^=91.3%, *P*<.001, prediction interval 0.13–4.79). Leave-one-out analysis again found that the Maya et al^41^ study was a large contributor to heterogeneity (Fig. 3B). Results with removal were not significant (OR 0.64, 95% CI, 0.30–1.37).

Two studies^38,40^ used slightly different ICD-10 codes to measure the outcome. Maya et al^41^ used intrapartum blood pressure measurements as their primary measure, with ICD-10 codes for missing values. Sensitivity analyses,^38,40,41^ propensity score matching,^38,41^ or statistical adjustment^40,41^ was used to reduce bias. Approach and covariate selection differed across studies.

Three studies^38,39,55^ with 182,538 pregnancies (46,095 exposed and 136,443 unexposed) were analyzed regarding risk of preeclampsia. Results were not significant (OR 1.05, 95% CI, 0.60–1.84) (Fig. 2C). There was significant, high heterogeneity, with a variance estimate of *τ*^*2*^*=*0.04 (95% CI, 0.00–2.33), *τ=*0.19, and *I*^2^=85.40% (*P*=.001). Preeclampsia was identified using ICD-10 codes. Two studies^38,39^ included prepregnancy exposures and used propensity score matching. Lewin et al^55^ did not attempt to limit bias using selection, matching, or statistical adjustment; the authors noted significant differences between exposure groups.

Eight cohorts from seven studies^35,38,40,41,55–57^ totaling 182,793 pregnancies (45,269 exposed and 137,514 unexposed) were analyzed to examine the association of exposure and risk of PTB (Fig. 2D). The estimated effect lacked significance (OR 1.01, 95% CI, 0.76–1.33). Between-study heterogeneity variance was estimated at *τ*^*2*^=0.06 (95% CI, 0.00–0.44, τ=0.25, prediction interval 0.52–1.95) with an *I*^2^ value of 64.5% (*P*=.006). The Lewin et al^55^ study was identified as highly influential to the pooled effect and the Imbroane et al^38^ study to between-study heterogeneity (*I*^2^) on influence analysis. In leave-one-out analysis, results after removal of the Imbroane et al^38^ study were not significant (OR 1.15, 95% CI, 0.97–1.36, *I*^2^=29.3%) (Fig. 3C). Graphical display of study heterogeneity diagnostics^60^ identified the Imbroane et al^38^ study as a potential outlier based on all three models tested and the Lewin et al^55^ study based on two of three. Subgroup analyses by exposure definition (before or during pregnancy) revealed no significant subgroup effects (between-group interaction *P*=.57). The diversity of approaches to address exposure indication, including using mixed exposure cohorts, precluded a reliable subgroup analysis based on this variable.

Although most studies defined PTB as delivery occurring between 22 and 37 weeks of gestation, two studies used different start points (weeks 20^40^ and 28^57^). Two^38,41^ included ICD-10 codes describing preterm delivery indication, but percentages by indication were not provided. All provided mean gestational age at delivery for the overall study population, but none provided details about proportions of deliveries occurring within different PTB categories (ie, extreme, very, or moderate-to-late). All but two^55,56^ reduced confounding through propensity score matching or adjustment. Exposure timing varied; only four studies^38,40,41,57^ intentionally included prepregnancy exposures.

Overall, study quality ranged from poor to high (Table 3). Potential bias stemmed from small sample size and comparability attributable to an inability to control for confounders and important covariates. Residual confounding was a concern because of study design. Only Maya et al^41^ provided detailed information about quality control methods. Exposure and outcome validation and limited descriptions of quality checks or missing data handling were noted reviewer concerns. Comparability was limited by imprecise documentation of exposure timing and variable definitions of key outcomes, with the exception of preeclampsia. The single RCT by Li et al^57^ was rated as having a low-to-moderate risk of bias using the RoB-2 framework. Reviewer concerns included the open-label approach, incomplete documentation regarding influence of attrition, and selective outcome reporting.

**Table 3.**
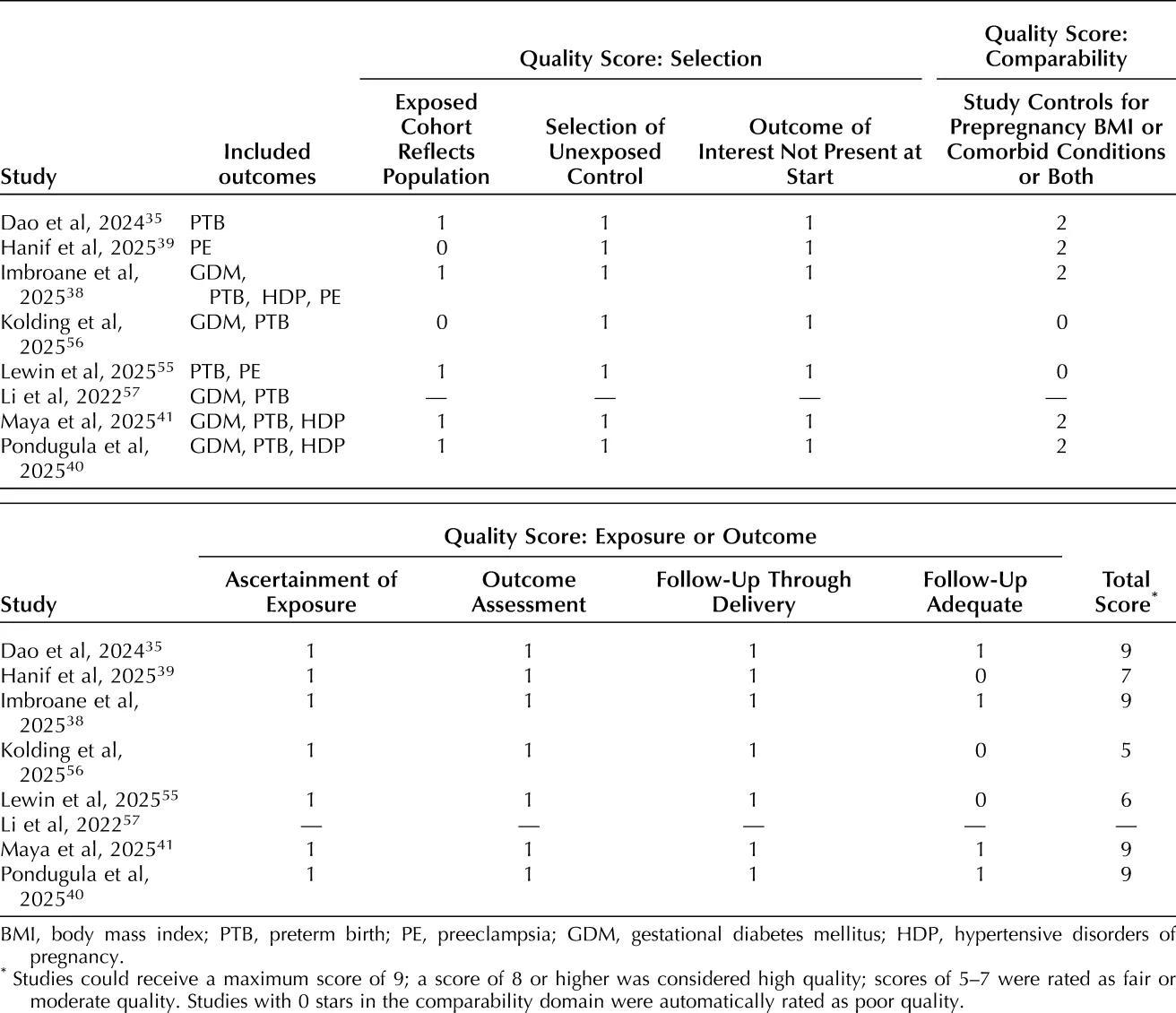
Key Outcomes and Study Quality as Measured by the Newcastle–Ottawa Scale

## DISCUSSION

In this systematic review and meta-analysis, we did not observe significant associations between peri-fertilization GLP-1 RA use (use within 3 years before fertilization and including early pregnancy) and GDM, HDP, preeclampsia, or PTB in our main analyses. However, the strength of these findings is limited by a small number of studies and substantial heterogeneity in methods. Leave-one-out sensitivity analyses suggest that peri-fertilization GLP-1 RA exposure may be associated with a decrease in odds of developing GDM when used before or during early pregnancy relative to no use. There was no evidence of variance in this analysis, and the OR prediction interval was below 1, suggesting the possibility for a true effect and reinforcing the need for continued research in this area. The short-term and long- term risks associated with GDM and poor glycemic control in the perinatal period more broadly have been well established.^12,13,16,61–63^ Despite this, the incidence of GDM continues to rise,^64^ and methods for reducing risk are limited.^65^ Although our results suggest a potential role for GLP-1–based therapies before pregnancy in GDM risk reduction, additional carefully designed studies are needed to better characterize this association and possible multigenerational risks.

Across the four phenotypes included in our final approach, we detected moderate-to-high heterogeneity (*I*^2^=65–91%), indicative of numerous methodologic differences across studies. One major difference between studies was the approach to address confounding. To address confounding by indication, for example, several studies selected exposed and unexposed comparators by indicating condition (eg, prepregnancy diabetes,^39,40,55^ weight loss management,^40^ or PCOS^57^), whereas others matched exposed and unexposed participants using a range of baseline health characteristics,^35,38,41^ including those same conditions. The only study to evaluate two separate exposure and control cohorts^40^ saw cohort differing associations, suggesting the importance of indication in the assessment of potential effects of GLP-1 RAs during the pregestational and early-gestational period.

Inadequate adjustment for confounders and inappropriate control selection could have contributed to lack of significance and wide CIs in our analyses. Lifestyle factors such as smoking or drug use and prior obstetric history, important for most pregnancy outcomes, were inconsistently adjusted for. Whether studies required the control group to be exposed to a non–GLP-1 RA antihyperglycemic medication also varied and could have affected comparability. Across studies, prematched GLP-1 RA users tended to have a greater number of risk factors for adverse pregnancy outcomes and were more likely to be multiparous. Studies that adjusted for relevant demographic and health differences were more likely to find statistically significant results. Two^38,40^ concluded that exposure lowered the odds of adverse pregnancy outcomes. One^41^ found that exposure increased risk, although the authors note that their population was older, less racially and ethnically diverse, more likely to have private insurance, and less likely to have BMI higher than 25 or to have public insurance than the general U.S. birthing population. For GDM, pooled results were significant when this study was omitted (Fig. 3C). Although our analysis did not find significant differences for HDP or preeclampsia, study-specific differences in demographics, ICD coding definitions, and results along with the existence of a plausible mechanism for HDP risk reduction—through weight loss and improved vascular remodeling^24,27^—recommend further research.

There was a lack of documentation regarding BMI measurement timing. According to available information, the BMI values used to control for obesity-related pregnancy risks likely reflected participants' postexposure BMI. Only one study^57^ provided data on participants' pre-exposure and postexposure weight and laboratory values. Because GLP-1 RAs induce weight loss, postexposure BMI likely represents a consequence of exposure rather than a baseline characteristic. Studies that match using this variable are effectively comparing GLP-1 RA–treated patients (who may have lost weight) with untreated individuals with similar BMI (who have not). Matching on an intermediate variable has the potential to induce bias or to obscure potential associations by conditioning on a mediator, potentially selecting for a control group participant with different metabolic risk or exposed participants with attenuated treatment response.

Although GLP-1 RAs may have differential effects on fertility and pregnancy outcomes, depending on the timing and duration of use, most included studies described difficulties obtaining exact exposure beginning and end dates, limiting our ability to perform reliable analyses stratified by timing. This variation in exposure timing represents a clear methodologic discrepancy in existing literature, one with implications for both this meta-analysis and the individual findings of the studies. Given that the major phenotypes altered by GLP-1 RA exposure (ie, weight, insulin sensitivity, and lipid homeostasis) fluctuate between prepregnancy and pregnancy and over the course of gestation in unexposed pregnancies and that these phenotypes themselves influence risk of adverse outcomes, the timing of exposure may result in distinct risk profiles for prepregnancy, fertilization, and gestational GLP-1 RA exposure. Combining exposure windows risks biasing results or concealing true effects that could be occurring in different exposure periods.

Specific medications and formulations included in exposure definitions also varied, which could have contributed to disparate individual study and meta-analyzed results. Decisions to limit to specific GLP-1 RAs or to perform sensitivity analyses to assess potential medication- or class-specific effects differed across studies. Older-generation GLP-1 RAs such as exenatide, used by Li et al*,*^57^ are known to be less effective than newer medications (eg, semaglutide).^40^ Glucose-dependent insulinotropic polypeptide–GLP-1 RA dual agonist medications such as tirzepatide were underrepresented and might have different associations.

Although our review focuses primarily on maternal outcomes, a discussion of fetal risk, particularly associations with major congenital malformations, is important and clinically relevant. Three included studies^14,39,55^ assessed congenital malformations. Both Dao et al^35^ and Lewin et al^55^ found similar rates of major congenital malformations between GLP-1 RA–exposed and unexposed groups. Hanif et al^39^ specifically examined fetal cardiac and renal anomalies and noted no statistically significant differences. These findings align with those from Morton and He,^34^ who found no increased risk after semaglutide exposure in a cohort of 150 type 2 diabetes–affected pregnancies (12 exposed), and Cesta et al*,*^14^ who concluded that there was no increased risk of major congenital malformations after early gestational exposure to GLP-1 RAs (n=938) compared with insulin exposure (n=5,078) in pregnancies of individuals affected by type 2 diabetes (adjusted relative risk 0.95, 95% CI, 0.72–1.26).

Although the results from this meta-analysis should be viewed as preliminary, our research has several strengths. First, the literature review was systematic, used multiple databases, and included primary literature from diverse geographic regions. Two reviewers, blinded to each other's responses, abstracted study data, and a third aided when collected data differed between them. We selected statistical methods that are reliable and appropriate for small-scale meta-analyses.^44,45,66^ In addition, we reported prediction intervals^58^ for each outcome and multiple measures of variance.

In addition to limitations imparted by the studies themselves, the small study number limited our ability to meta-analyze some pregnancy outcomes. Significant heterogeneity affected our results, demonstrated by the leave-one-out analysis for GDM. Although heterogeneity estimates and typical thresholds of significance may be unstable when the sample size is small,^66^ we are confident in our heterogeneity and sensitivity analysis results given the number of study design differences we documented, including differences in exposure and outcome ascertainment. Finally, we pooled unadjusted and adjusted effect estimates in some analyses, which could introduce heterogeneity and bias in these analyses as a result of residual confounding and decrease validity of the findings. However, the small number of studies and with many of the included studies providing unadjusted effect estimates only, we were unable to perform reliable sensitivity analyses restricted to adjusted estimates only.

As more research becomes available, this meta-analysis should be updated. To improve comparability and interpretation of results, studies should attempt to standardize exposure timing and ascertainment methods. For studies including both prepregnancy and gestational exposure, we suggest keeping the prepregnancy exposure period to a maximum of 24 months before fertilization and excluding participants on the basis of clearly defined exposure end points to avoid ambiguity regarding which stages of fetal development were encompassed in analysis. Additional stratified analyses to assess potential differential effects based on GLP-1 RA timing (ie, prefertilization vs gestational, short-term vs long-term use, time from last prescription to fertilization), formulation, and dosage are also recommended.

For individuals with prepregnancy use, GLP-1 RA–induced changes in indication (eg, reduction in BMI or improved blood glucose) need consideration because these variables influence pregnancy risk. When possible, we suggest using pre-exposure values for matching. Studies using BMI after or during GLP-1 RA exposure should carefully consider their statistical methods and aim to perform additional analyses investigating potential mediating effects. Careful attention should be paid to potential adverse effects of GLP-1 RA discontinuation, for example, weight rebound,^28^ which might attenuate or negate the benefit of entering pregnancy at a lower BMI. Potential alterations to gestational weight gain rate and timing should also be attended to because each has uncertain implications for pregnancy health.^67,68^ Future research might explore whether disparate associations with adverse pregnancy outcomes are dependent on the amount of prepregnancy change, timing of exposure compared with fertilization, or whether exposure-related changes are sustained.

When possible, outcome definitions including multiple measures—eg, laboratory values along with ICD-10 codes—are recommended. Studies on broad phenotypes such as HDP and PTB might consider including proportions by subtype and stratifying analyses if sample size permits. For PTB, reporting the percentage of births within each World Health Organization category^69^ or including spontaneous compared with indicated PTB as a covariate or stratification variable has clinical utility because the pathologic mechanisms that lead to either are disparate.^70,71^ Future research should continue to assess gestational weight gain, cesarean delivery, and miscarriage.

Information on major confounders and factors related to GLP-1 RA access should be collected, reported, and used during patient selection and analysis. This should include maternal age, race and ethnicity, smoking status, comorbidities, prepregnancy hemoglobin A_1C_, use of non–GLP-1 RA antihyperglycemic medications, and obstetric history. For studies exploring multiple outcomes or GLP-1 RA indications, broad matching during cohort selection or stratification should be performed first; later adjustment for confounders should be specific to each outcome.

This meta-analysis highlights a critical gap in the current evidence base. These recommendations aim to assist with future efforts within the scientific community to build on the findings of this meta-analysis; to optimize counseling and clinical management of individuals with overweight, obesity, or type 2 diabetes; and ultimately to improve pregnancy outcomes for individuals with prepregnancy use of GLP-1 RA medications. Pregnancy registries and electronic health record–based datasets should consider collecting GLP-1 RA use in future data collection because this could provide a clear pathway for future research and application of these recommendations.

This systematic review and meta-analysis demonstrates that, to date, the literature on GLP-1 RA exposure before and in early pregnancy is small and heterogeneous. Based on limited studies, our analysis demonstrates no consistent associations with adverse obstetric outcomes, although exploratory analyses suggest that exposure to GLP-1 RAs may lower the odds of developing GDM for women with indications for GLP-1 RA use before pregnancy. Further research is necessary to explore these hypotheses-generating results, to ascertain whether duration and timing of exposure or magnitude of weight loss influences the strength of associations, and to identify possible associations with other pregnancy outcomes. As GLP-1 RA use rises, understanding the therapeutic potential and specific risks of these medications in the peri-fertilization period is critical and clinically urgent.

## Supplementary Material
