# Supplementary material for: Glucagon-Like Peptide-1 Receptor Agonists and Risk of Adverse Maternal Pregnancy Outcomes: A Systematic Review and Meta-analysis
Source: Obstet Gynecol. 2026 Jul 2;148(4):220–36. doi: 10.1097/AOG.0000000000006363 (PMC13489104; doi:10.1097/AOG.0000000000006363)
Supplement: Supplementary file 1 [file ong-148-0220-s001.pdf]

Appendix I: Full Search Strategy by Database

EMBASE: ('glp-1'/exp OR 'glp-1' OR 'glp1' OR 'glp-1*' OR 'glp1*' OR 'glucagon-like peptide-1'/exp OR 'glucagon-like peptide-1' OR 'glucagon like peptide 1'/exp OR 'glucagon like peptide 1' OR 'semaglutide'/exp OR 'semaglutide' OR 'liraglutide'/exp OR 'liraglutide' OR 'dulaglutide'/exp OR 'dulaglutide' OR 'exenatide'/exp OR 'exenatide' OR 'lixisenatide'/exp OR 'lixisenatide' OR 'tirzepatide'/exp OR 'tirzepatide' OR 'orlistat'/exp OR 'orlistat' OR 'ozempic'/exp OR 'ozempic' OR 'wegovy'/exp OR 'wegovy' OR 'saxenda'/exp OR 'saxenda' OR 'trulicity'/exp OR 'trulicity' OR 'byetta'/exp OR 'byetta' OR 'bydureon'/exp OR 'bydureon' OR 'adlyxin'/exp OR 'adlyxin' OR 'mounjaro'/exp OR 'mounjaro' OR 'glucagon-like peptide 1'/exp OR 'glucagon-like peptide 1' OR 'glucagon-like peptide-1 receptor agonists'/exp OR 'glucagon-like peptide-1 receptor agonists') AND ('pre-eclampsia'/exp OR 'pre-eclampsia' OR 'hypertension, pregnancy-induced'/exp OR 'hypertension, pregnancy-induced' OR 'eclampsia'/exp OR 'eclampsia' OR 'hellp syndrome'/exp OR 'hellp syndrome' OR 'diabetes, gestational'/exp OR 'diabetes, gestational' OR 'pregnancy outcome'/exp OR 'pregnancy outcome' OR 'maternal health'/exp OR 'maternal health' OR 'maternal morbidity'/exp OR 'maternal morbidity' OR (('maternal'/exp OR maternal) AND ('morbidity'/exp OR morbidity)) OR gdm OR 'gestational hyperglycemia'/exp OR 'gestational hyperglycemia' OR (gestational AND ('hyperglycemia'/exp OR hyperglycemia)) OR 'maternal outcome'/exp OR 'maternal outcome' OR (('maternal'/exp OR maternal) AND ('outcome'/exp OR outcome)) OR 'hypertensive disorders of pregnancy'/exp OR 'hypertensive disorders of pregnancy' OR (hypertensive AND ('disorders'/exp OR disorders) AND ('pregnancy'/exp OR pregnancy)) AND ('pre-pregnancy' OR 'prepregnancy'/exp OR 'prepregnancy' OR 'preconception'/exp OR 'preconception' OR 'pre-conception' OR 'before pregnancy' OR 'prior to pregnancy' OR 'preconceptional' OR 'pregravid' OR 'pre-gestational') AND 'human'/de

PubMed: (("GLP-1" OR "GLP1" OR "GLP-1*" OR "GLP1*" OR "glucagon-like peptide-1" OR "glucagon like peptide 1" OR "semaglutide" OR "liraglutide" OR "dulaglutide" OR "exenatide" OR "lixisenatide" OR "tirzepatide" OR "orlistat" OR "ozempic" OR "wegovy" OR "saxenda" OR "trulicity" OR "byetta" OR "bydureon" OR "adlyxin" OR "mounjaro" OR "Glucagon-Like Peptide 1"[Mesh] OR "Hypoglycemic Agents"[Mesh] OR "Glucagon-Like Peptide-1 Receptor Agonists"[MeSH Terms]) AND ((("Pre-Eclampsia"[Mesh]) OR "Hypertension, Pregnancy-Induced"[Mesh]) OR "Diabetes, Gestational"[Mesh]) OR "Eclampsia"[Mesh]) OR "HELLP Syndrome"[Mesh]) OR "Pregnancy Outcome"[Mesh]) OR "Maternal Health"[Mesh]) OR (maternal morbidity)) OR (GDM)) OR (gestational hyperglycemia)) OR (maternal outcome)) OR (hypertensive disorders of pregnancy)) OR (gestational diabetes) OR (diabetes, pregnancy-induced)) AND ("pre-pregnancy" OR "prepregnancy" OR "preconception" OR "pre-conception" OR "before pregnancy" OR "prior to pregnancy" OR "preconceptional" OR "pregravid" OR "pre-gestational")

Hattler E, Schluter H, Greene C, Adgent MA, Sundermann AC, Jasper EA. Glucagon-like peptide-1 receptor agonists and risk of adverse maternal pregnancy outcomes: a systematic review and meta-analysis. Obstet Gynecol 2026;148.
The authors provided this information as a supplement to their article.
©2026 The Authors.
