# Supplementary material for: Glucagon-Like Peptide-1 Receptor Agonists and Risk of Adverse Maternal Pregnancy Outcomes: A Systematic Review and Meta-analysis
Source: Obstet Gynecol. 2026 Jul 2;148(4):220–36. doi: 10.1097/AOG.0000000000006363 (PMC13489104; doi:10.1097/AOG.0000000000006363)
Supplement: Supplementary file 2 [file ong-148-0220-s002.pdf]

NOTICE: This document contains correspondence generated during peer review and subsequent revisions but before transmittal to production for composition and copyediting:

- Comments from the reviewers and editors (email to author requesting revisions)
- Response from the author (cover letter submitted with revised manuscript)*

**The corresponding author has opted to make this information publicly available.*

Personal or nonessential information may be redacted at the editor's discretion.

Questions about these materials may be directed to the *Obstetrics & Gynecology* editorial office: obgyn@greenjournal.org.

Date: 03/10/2026
To: "Elizabeth Jasper" [redacted]
From: "The Green Journal" em@greenjournal.org
Subject: Your Submission ONG-26-150

03/10/2026

RE: ONG-26-150: GLP-1 receptor agonists and risk of adverse maternal pregnancy outcomes: a systematic review and meta-analysis

Dear Dr. Jasper:

Thank you for sending us your work for consideration for publication in Obstetrics & Gynecology. Your manuscript has been reviewed by the Editorial Board and by special expert referees. The Editors would like to invite you to submit a revised version for further consideration.

If you wish to revise your manuscript, please read the following comments submitted by the reviewers and Editors. Each point raised requires a response, by either revising your manuscript or making a clear argument as to why no revision is needed in the cover letter.

To facilitate our review, we prefer that the cover letter you submit with your revised manuscript include each reviewer and Editor comment below, followed by your response. That is, a point-by-point response is required to each of the EDITOR COMMENTS (if applicable), REVIEWER COMMENTS, and STATISTICAL EDITOR COMMENTS (if applicable) below. The revised manuscript should indicate the position of all changes made. Please use the "track changes" feature in your document (do not use strikethrough or underline formatting). Upload the tracked-changes version when you submit your revised manuscript.

Your submission will be maintained in active status for 21 days from the date of this letter. If we have not heard from you by 03/31/2026, we will assume you wish to withdraw the manuscript from further consideration.

EDITOR COMMENTS:

Please note the following:

* Help us reduce the number of queries we add to your manuscript after it is revised by reading the Revision Checklist at https://journals.lww.com/greenjournal/Documents/RevisionChecklist_Authors.pdf and making the applicable edits to your manuscript.

* As of January 2024, only certain article types will appear in the print version of the journal. All accepted articles will continue to publish online. All articles will be indexed in PubMed as an official article of Obstetrics & Gynecology. Additional information is available in the Instructions for Authors (https://journals.lww.com/greenjournal/Pages/InformationforAuthors.aspx#II).

* Figure 1: OK
* Figures 2 and 3: Please include a legend for each figure. Please upload each figure separately as a high-resolution figure file on Editorial Manager.

REVIEWER COMMENTS:

Reviewer #1: 1.The manuscript lacks a clear operational definition of the periconception period, which represents a notable methodological gap. Given that the periconception window has been variously defined in the literature—spanning anywhere from weeks before conception through early embryonic development and implantation—the authors' failure to explicitly delineate the boundaries of this exposure period introduces ambiguity regarding which developmental stages are encompassed the studies in their analysis. They briefly mention in their manuscript some of the variations of that definition by one study.

2. Including a statement about GLP-1 receptor agonist use in pregnancy registries would be valuable for this meta-analysis because it acknowledges a critical gap in the current evidence base and provides a pathway for future data collection.

3. Overall, the manuscript is well written and methodologically sound, with a clear and logical structure that facilitates reader comprehension. The authors have addressed an important and timely clinical question. With the minor revisions outlined above—particularly the inclusion of an explicit operational definition of the periconception period and acknowledgment of ongoing pregnancy registries—this manuscript would make a valuable contribution to the literature.

Reviewer #2: This is a well written article on this topic.
1. From an obstetric perspective, one of the most common and clinically relevant questions is whether an exposure will affect the fetus. While this review appropriately focuses on maternal outcomes, was there sufficient information across the included studies to comment on fetal risks, particularly congenital anomalies? Even if the available data are limited, a brief summary or dedicated paragraph addressing fetal safety would substantially strengthen the manuscript and enhance its clinical applicability.

2. Lines 316-318 state: "As GLP-1RAs induce weight loss, post-exposure BMIs likely represent a consequence of exposure rather than a baseline characteristic." Could you elaborate on this point? If the exposure occurs pre-pregnancy or in the first trimester, the BMI at that time would typically be considered the baseline value used for obstetric risk stratification and counseling. Clarifying how and when BMI was measured relative to exposure would help readers interpret whether BMI is being treated as a confounder, a mediator, or an effect of the medication.

Reviewer #3: Authors present systematic review and metanalysis of GLP1 periconception exposure on maternal obstetric outcomes. This is a careful statistical synthesis of highly heterogeneous/confounded literature with small numbers. The manuscript is generally well-written and thoughtful. My comments focus on interpretability of outcomes.

Introduction: well-written, and clearly sets up the knowledge gap and justifications for this manuscript

Methods:
- The authors use MOOSE guidelines, which is appropriate given predominance of observational studies. Could consider also reporting with PRISMA 2020 standards or state why this was not used.
- Adjusted effect estimates are pooled with unadjusted estimates when necessary - this could introduce interpretability challenges. Should consider sensitivity analyses restricting to adjusted estimates only, and discuss the implications of mixing effect types. If not performed, should justify why.
- Given biologically distinct implications of preconception vs intra-pregnancy exposure, similarly should consider analyses stratified by exposure timing. If not, should justify why.

Discussion:
- Leave-one-out analysis for GDM- main pooled estimate was null with high heterogeneity and results achieved statistical significance after exclusion of a study. Language that this could be a true effect (line 263-268) should be softened to exploratory and hypothesis generating.
- Similarly, the conclusion should also not be anchored to this post-hoc finding (line 409-411) - rather conclusion should be that "there are no consistent associations to adverse obstetric outcomes" as the main headline and exploratory analyses suggest reduced GDM risk - instead of "it might be protective against GDM" as the sole message.
- The "Recommendations for Future Research" section is well written and welcome commentary

STATISTICAL EDITOR COMMENTS: The objective of this meta-analysis was to evaluate associations between periconception GLP-1 exposure and maternal pregnancy complications. Overall, this manuscript was well written and clear. The analysis plan was also well written and the justification for using Hartung-Knapp method was clear and appropriately used. There were a few details in the results that need clarification or need to be checked.

Lines 97-98: Interesting that authors were open to including unpublished research that have not been peer reviewed. However, since it seems that only published peer reviewed research ended up being included in this meta-analysis, the authors should consider rewording these statements.

Lines 112-124: Results included prediction intervals, but these were not described in the analysis plan. Please also describe that prediction intervals were also calculated. Additionally, please report statistical significance level considered in this analysis.

Lines 140-142: Not clear how these numbers correspond to the numbers in Table 1. Would expect n=42242 exposed and n=221442 unexposed.

Line 231: Not clear if graphical displays for evaluation of heterogeneity were previously described or presented in the results.

Table 2: For "Variables adjusted and/or matched for", was this for any adjusted analysis mentioned in the original study, or for outcomes included in this meta-analysis? For Dao et al., it is a little confusing given that only preterm birth was included from this study, but it specifies that there were not adjustments made while also listing out covariates.

Table 3: Please also describe how to interpret what the scores mean.

Figure 2: Some of these prediction intervals extend beyond the x-axis. Please update limits of the x-axis.

Figure 3: This analysis is very helpful in evaluating the robustness of these results. To ease the review of these results, please consider removing column for Tau. Not clear if both Tau^2 and Tau need to both be presented in these forest plots.

--
Sincerely,

The Editors of Obstetrics & Gynecology

In compliance with data protection regulations, you may request that we remove your personal registration details at any time. (Use the following URL: https://www.editorialmanager.com/ong/login.asp?a=r). Please contact the publication office if you have any questions.

March 23, 2026

Obstetrics and Gynecology

Dear Editors,

We are pleased to submit a revised version of our manuscript entitled "*GLP-1 receptor agonists and risk of adverse maternal pregnancy outcomes: a systematic review and meta-analysis*" for consideration for publication in *Obstetrics & Gynecology*. This systematic review and meta-analysis synthesizes current evidence on glucagon-like peptide-1 receptor agonist (GLP-1RA) exposure prior to conception and in early pregnancy, examining associations with gestational diabetes, hypertensive disorders of pregnancy, preeclampsia, and preterm birth. Our review includes eight studies comprising 186,598 pregnancies, over 47,000 of which had exposure to GLP-1RAs in the periconception period. There were no significant associations between GLP-1Ras and adverse pregnancy outcomes in our analyses, though, in exploratory analyses we found individuals on GLP-1RAs around the time of conception may have a reduced risk of gestational diabetes compared to those not on the medications. We believe these findings will be of immediate clinical relevance to your readership.

We conducted a comprehensive search of Embase and PubMed (including MEDLINE and ClinicalTrials.gov results) through November 16, 2025, adhering to the MOOSE reporting guidelines. We separately searched ClinicalTrials.gov but this search did not yield additional relevant results. We have maintained transparency by providing detailed methodology including our full search strategy, flow diagram, and statistical analysis methods. The study protocol and statistical analysis plan were finalized before data extraction began. All authors meet ICMJE criteria for authorship and have approved this submission. We have no conflicts of interest to report. The manuscript is not under consideration elsewhere and we have no conflicts of interest to declare. An early draft of our abstract was accepted and will be presented as a poster at the Society for Reproductive Investigation Annual Meeting in San Juan, Puerto Rico on March 28, 2026. The research did not involve human subjects and therefore did not require IRB approval.

We thank the editor and reviewers for their helpful comments and feel they have greatly improved the quality of the manuscript. In addition to making the requested edits to the final manuscript, we have responded to all reviewer comments in full below. We also included a legend for Figure 2 and 3 in the manuscript and provided separate high-resolution PNG files for the figures. Thank you for consideration of this submission. We look forward to your response. If you should need any additional information, please contact me at elizabeth.jasper@vumc.org.

Sincerely,

Elizabeth Jasper

Elizabeth A. Jasper, PhD, MS
Assistant Professor
Division of Quantitative and Clinical Sciences,
Department of Obstetrics and Gynecology
Department of Biomedical Informatics
Vanderbilt University Medical Center
[redacted]

[redacted] [redacted]

Editor comments

Figure 1: OK

Figures 2 and 3: Please include a legend for each figure. Please upload each figure separately as a high-resolution figure file on Editorial Manager.

We have edited the images to include a legend for each figure.

Reviewer comments

Reviewer #1:

Overall, the manuscript is well written and methodologically sound, with a clear and logical structure that facilitates reader comprehension. The authors have addressed an important and timely clinical question. With the minor revisions outlined (below)—particularly the inclusion of an explicit operational definition of the periconception period and acknowledgment of ongoing pregnancy registries—this manuscript would make a valuable contribution to the literature.

The manuscript lacks a clear operational definition of the periconception period, which represents a notable methodological gap. Given that the periconception window has been variously defined in the literature—spanning anywhere from weeks before conception through early embryonic development and implantation—the authors' failure to explicitly delineate the boundaries of this exposure period introduces ambiguity regarding which developmental stages are encompassed the studies in their analysis. They briefly mention in their manuscript some of the variations of that definition by one study.

We chose to keep the operational definition of the periconception period, especially the timing before conception, broad to ensure we captured all the existing literature on this topic. We did this by using keywords for preconception (prepregnancy, periconception, etc.). Our decision was based on a non-systematic search prior to our systematic review, which demonstrated variability in exposure time points across studies. Identified literature from the systematic review resulted in an operational definition of the periconception period as within the three years before conception through pregnancy (primarily into the first trimester, though some studies included exposures in the second). We have edited our methods (lines 71 – 72 & 91 – 92) and discussion (lines 261 – 262, 323 – 347) to make our definition and rationale explicit, as well as add discussion of variation in exposure timing and the potential impact of pooling exposure time periods. We have additionally strengthened our recommendations about exposure timing (lines 400 – 404) as we agree with the reviewer that variability in this area could have a major impact on risk of fetal and maternal outcomes.

Including a statement about GLP-1 receptor agonist use in pregnancy registries would be valuable for this meta-analysis because it acknowledges a critical gap in the current evidence base and provides a pathway for future data collection.

We agree with the reviewer and thank them for this suggestion. We have added a sentence (lines 444 – 446) stating that pregnancies registries should consider collecting data on GLP-1 receptor agonist use, as this would pave the way for future research and application of these recommendations.

[redacted]

[redacted]

Reviewer #2:
This is a well written article on this topic.
From an obstetric perspective, one of the most common and clinically relevant questions is whether an exposure will affect the fetus. While this review appropriately focuses on maternal outcomes, was there sufficient information across the included studies to comment on fetal risks, particularly congenital anomalies? Even if the available data are limited, a brief summary or dedicated paragraph addressing fetal safety would substantially strengthen the manuscript and enhance its clinical applicability.
We chose to focus our review primarily on maternal outcomes given reassuring data from several peer-reviewed studies (Cesta et al., 2024, Dao et al., 2024, Morton et al., 2025) on the association between GLP-1 exposure and major congenital malformations alongside conflicting data on maternal outcomes. In addition, our initial non-systematic search found a pre-print of a meta-analysis that focused on fetal outcomes (Armstrong et al., 2025), which found no statistically significant differences for risk of major congenital malformations. Their findings on major congenital malformations were consistent with other recent manuscripts on this topic. We hoped to include gestational weight gain, spontaneous abortion, miscarriage, large for gestational age, and small for gestational age deliveries in our analysis. However, low study number and/or heterogeneity in outcome definitions and assessment precluded meta-analysis of these outcomes. We agree that a dedicated paragraph addressing fetal safety is an important addition to this manuscript and have added one to the discussion section (lines 362 – 373).

Lines 316-318 state: "As GLP-1RAs induce weight loss, post-exposure BMIs likely represent a consequence of exposure rather than a baseline characteristic." Could you elaborate on this point? If the exposure occurs pre-pregnancy or in the first trimester, the BMI at that time would typically be considered the baseline value used for obstetric risk stratification and counseling. Clarifying how and when BMI was measured relative to exposure would help readers interpret whether BMI is being treated as a confounder, a mediator, or an effect of the medication.
The timing of BMI measurement for the included studies was often ambiguous, though as the reviewer state's prepregnancy BMI is critical for obstetric risk stratification and counseling. The choice of which BMI measurement is used has study design considerations. We've elaborated on this point (lines 317 – 322) in accordance with this reviewer's suggestion. Using BMI measures prior to GLP-1RA exposures in the exposed group may be more appropriate for comparison with unexposed individuals as the alternative is to use post-exposure BMI (which may be a consequence of the medication or a mediator).

Reviewer #3:
Authors present systematic review and metanalysis of GLP1 periconception exposure on maternal obstetric outcomes. This is a careful statistical synthesis of highly heterogeneous/confounded literature with small numbers. The manuscript is generally well-written and thoughtful. My comments focus on interpretability of outcomes.
Introduction: well-written, and clearly sets up the knowledge gap and justifications for this manuscript
Methods:
The authors use MOOSE guidelines, which is appropriate given predominance of observational studies. Could consider also reporting with PRISMA 2020 standards or state why this was not used.
We appreciate this reviewer's suggestion. Per journal requirements, use of either MOOSE or PRISMA reporting guidelines is acceptable. We have chosen to proceed using the MOOSE reporting guidelines as most of the included studies were observational in nature.

[redacted] [redacted]

Adjusted effect estimates are pooled with unadjusted estimates when necessary - this could introduce interpretability challenges. Should consider sensitivity analyses restricting to adjusted estimates only, and discuss the implications of mixing effect types. If not performed, should justify why.
We acknowledge the reviewer's concern about the impact of pooling unadjusted and adjusted effect estimates in our analyses and agree with the reviewer that this introduces some challenges in interpretability and validity of results. However, many of the included studies did not provide adjusted effect estimates. Combined with the small number of studies, this precluded reliable sensitivity analyses where we restricted to adjusted effect estimates. We have added these points to the strengths and limitations section of the discussion (lines 388 – 393), stating that this could introduce additional heterogeneity, bias results due to residual confounding, and decrease validity of the findings, but we were unable to perform analyses restricted to studies with adjusted effect estimates due to the small number of studies.

Given biologically distinct implications of preconception vs intra-pregnancy exposure, similarly should consider analyses stratified by exposure timing. If not, should justify why.
Many of the studies included in our analysis had difficulties obtaining exact exposure beginning and endpoints. Even if study authors chose to focus on intra-pregnancy (or preconception) exposures, their population invariably included those with exposures falling within the other exposure period. For this reason, and the small study number ($k \leq 4$) for most of our analyzed outcomes, we were unable to stratify our analyses by exposure timing. Per reviewer 1 and reviewer 3's suggestion, we have also reorganized the discussion and elaborated on the exposure timing and its impact on results (lines 323 – 347).

Discussion:
Leave-one-out analysis for GDM: main pooled estimate was null with high heterogeneity and results achieved statistical significance after exclusion of a study. Language that this could be a true effect (line 263-268) should be softened to exploratory and hypothesis generating. Similarly, the conclusion should also not be anchored to this post-hoc finding (line 409-411), rather conclusion should be that "there are no consistent associations to adverse obstetric outcomes" as the main headline and exploratory analyses suggest reduced GDM risk, instead of "it might be protective against GDM" as the sole message.
We appreciate this reviewer's suggestions and have made edits to the manuscript (lines 266 – 269, 449 – 453) to refine and soften the language used in the results and discussion. We have also edited the precis to focus on the lack of association between GLP-1 receptor agonist exposure and adverse pregnancy outcomes.
The "Recommendations for Future Research" section is well written and welcome commentary.

STATISTICAL EDITOR COMMENTS:
The objective of this meta-analysis was to evaluate associations between periconception GLP-1 exposure and maternal pregnancy complications. Overall, this manuscript was well written and clear. The analysis plan was also well written and the justification for using Hartung-Knapp method was clear and appropriately used. There were a few details in the results that need clarification or need to be checked.

Lines 97-98: Interesting that authors were open to including unpublished research that have not been peer reviewed. However, since it seems that only published peer reviewed research ended up being included in this meta-analysis, the authors should consider rewording these statements.
Given the recency of this field of research and the risk that exclusion of "grey" literature from analysis might lead to exaggerated effect estimates (McAuley et al., 2000), we made the *a priori* decision that we would include grey literature meeting all other inclusion criteria and included an appropriate and well-documented

methodological/analysis plan. As this reviewer indicates, we ultimately did not include any non-peer reviewed studies in our analysis as our systematic literature review did not identify any grey literature that fit all other inclusion criteria. We have reworded lines 96 – 99.

Lines 112-124: Results included prediction intervals, but these were not described in the analysis plan. Please also describe that prediction intervals were also calculated. Additionally, please report statistical significance level considered in this analysis.
We have added description of the analytical strategy, including the prediction interval, in our methods section. We have also reported the 95% statistical significance level considered in our analysis (lines 112 – 128).

Lines 140-142: Not clear how these numbers correspond to the numbers in Table 1. Would expect n=42,242 exposed and n=221,442 unexposed.
We appreciate the reviewer bringing this to our attention. As they stated, there was a mismatch between the numbers presented in Table 1 and those documented in text. The text numbers are correct. The discrepancy was due to inputting the total sample size from each study, as opposed to the number of pregnancies. We also did not limit values for certain studies to the cohorts we utilized. We have renamed this column as "pregnancies analyzed (# pregnancies exposed)" and edited it accordingly. This column is now the number of pregnancies analyzed in the study that we have used for the meta-analyses. Imbroane et al 2025 now specifies 6,676 pregnancies, half of which are exposed as opposed to 8,534. This was to match the cohort we used from their study, which used a 12 month preconception exposure window. This is also why the values for Kolding et al 2025 changed (as in our analyses we used the smaller comparison cohort of insulin users). Finally, Lewin et al 2025 previously listed the number of individuals in the study. However, they used all pregnancies from these individuals in their analysis. We have reviewed all numbers and totals in Table 1 to ensure accuracy. We have also added a footnotes to the Imbroane, Kolding, and Lewin entries in table one to state which cohorts were used and that Lewin et al 2025 allowed all individuals pregnancies in a defined period as opposed to other studies where a single pregnancy from each person was utilized.

Line 231: Not clear if graphical displays for evaluation of heterogeneity were previously described or presented in the results.
We chose to reserve GOSH diagnostics and subgroup analysis for outcomes representing more than five studies in order to have adequate power. Preterm birth was the only outcome with k > 5 studies. We have added specifications about the preterm birth GOSH analysis to the methods section (lines 123 – 126).

Table 2: For "Variables adjusted and/or matched for", was this for any adjusted analysis mentioned in the original study, or for outcomes included in this meta-analysis? For Dao et al., it is a little confusing given that only preterm birth was included from this study, but it specifies that there were not adjustments made while also listing out covariates.
The column "Variables adjusted and/or matched for" in Table 2 should only include adjustments relative to the outcomes that were meta-analyzed. We appreciate this reviewer bringing our attention to the row for Dao et al., which included erroneous details on adjustments made for outcomes we ended up not including due to lack of adequate study number. We have edited this row and reviewed the entirety of Table 2 to ensure all details are correct.

Table 3: Please also describe how to interpret what the scores mean.

We have added details on scoring to the table and lines 128 – 130.

Figure 2: Some of these prediction intervals extend beyond the x-axis. Please update limits of the x-axis.
We have edited the x-axis limits of both Figure 1 and Figure 2 to ensure prediction intervals do not extend beyond axis limits.

Figure 3: This analysis is very helpful in evaluating the robustness of these results. To ease the review of these results, please consider removing column for Tau. Not clear if both Tau^2 and Tau need to both be presented in these forest plots.
We have edited Figure 3 to remove the column for tau.
